# Exploring Substituted Tetrazoloquinazoline: Biological Activities, Molecular Docking Analysis, and Anti-Breast Cancer MCF7/HER2 Effects

**DOI:** 10.1155/2024/6952142

**Published:** 2024-08-28

**Authors:** Neni Frimayanti, Ihsan Ikhtiarudin, Rahma Dona, Rahul Oktarizal, Aprilia Cindy Nurfatimah

**Affiliations:** ^1^ Department of Pharmacy Sekolah Tinggi Ilmu Farmasi Riau, Jalan Kamboja, Simpang Baru, Pekanbaru 28293, Indonesia; ^2^ Department of Chemistry Faculty of Mathematics and Natural Science Universitas Riau, Pekanbaru, Indonesia

## Abstract

Breast cancer is a condition where breast tissue cells grow uncontrollably. Various natural and synthesized compounds, such as quinazoline, have been studied for their potential as anticancer agents. Quinazoline derivatives have shown diverse bioactivities, including antimalarial, antifungal, antimicrobial, and anticancer properties. This research aims to synthesize substituted tetrazoloquinazoline and evaluate its potential as an anticancer agent using molecular docking studies with the Molecular Operating Environment (MOE) software. Furthermore, molecular dynamic was also performed to analyze the binding stability of this protein-ligand complex. Additionally, the physicochemical and pharmacokinetic properties of quinazoline compounds were assessed using the website https://www.swissadme.ch. The cytotoxic activity of the compounds was evaluated using the MTT assay. The docking results revealed that substituted tetrazoloquinazoline exhibited a significantly different range of binding free energy compared to the positive control. Moreover, the substituted tetrazoloquinazoline compounds comply with Lipinski's Rule of Five (Ro5), indicating that they are easily absorbable and have good permeability. The cytotoxic activity of the compounds was found to have an IC_50_ value of >1000 ppm, classifying them as noncytotoxic. It therefore paved the way for the discovery of promising next-generation drugs against breast cancer.

## 1. Introduction

Breast cancer is a common cancer that primarily affects women and is a leading cause of death. Data compiled by the World Health Organization (WHO) reveal that approximately 2.1 million women are diagnosed with breast cancer each year [1]. Breast cancer exhibits different characteristics based on receptors, including the estrogen receptor (ER), progesterone receptor (PR), and human epithelial receptor 2 (HER2). These characteristics allow breast cancer cell lines to be divided into five groups: luminal A, luminal B, positive HER2, triple negative A, and triple negative B. The breast cancer cell lines MCF7, SK-Br3, and MDA-MB231 represent luminal subtypes A, HER2, and triple negative B, respectively. Each group responds differently to therapy due to variation in their receptors [2–4]. When receptors are present in breast cancer cells, they can bind to a ligand and produce specific effects if the ligand matches with the breast cancer cell receptor. If the binding of the ligand to the receptor inhibits the growth of breast cancer cells, a substance containing the ligand can be utilized for therapy. Therefore, it is crucial to identify and develop new ligands that can effectively impede cancer cell growth. Quinazoline is an example of such a compound [5].

Quinazoline derivatives are a promising group of N-containing heterocyclic compounds for drug research due to their diverse biological activities, including analgesic, anti-inflammatory, antihypertensive, antimicrobial, antiviral, anticancer, antioxidant, and anticonvulsant activities [6–11]. In addition, compounds based on quinazoline have also shown great promise as chemotherapeutic agents with significant effectiveness against different types of tumors [12–14].

Currently, quinazoline has also contributed to the development of numerous clinically used drugs, such as afatinib, albaconazole, alfuzosin, balaglitazone, barasertib, cediranib, sotrastaurin, elinogrel, fluconazole, mubritinib, ribavirin, and itraconazole. The demand for novel chemical compounds and biologically active substances is constantly rising, posing a challenge to medicinal chemistry researchers to develop effective drugs within shorter timeframes. However, the synthesis of new molecules with potent activity, high selectivity, drug-likeness, and favorable pharmacokinetic properties remains an exciting endeavor. Thus, various heterocyclic ring structures are being designed to enhance potency and binding efficiency with receptors through structural modifications.

Several in silico investigations have been conducted on quinazolines [15]. However, there is a lack of extensive research on quinazoline compounds as anticancer agents, particularly for breast cancer. Additionally, there are limited studies on in silico methods such as molecular docking and ADME calculations combined with pharmacological networks for breast cancer inhibitors. Therefore, the purpose of this study is to synthesize substituted tetrazoloquinazoline and conduct in silico study to predict the activity of this compound. Subsequently, the compound will be tested in vitro on MCF-7/HER2 cells which are widely recognized by researchers worldwide.

## 2. Materials and Methods

### 2.1. General Method for Synthesis of Substituted Tetrazoloquinazolines

**Figure 1:**
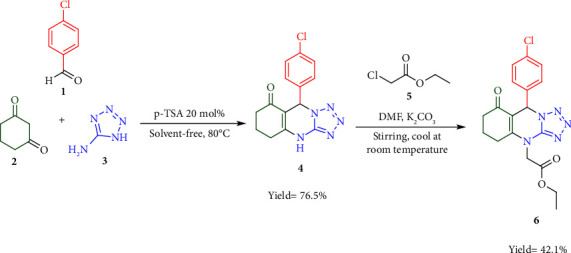
Synthesis route of substituted tetrazoloquinazoline.

The synthesis route of substituted tetrazoloquinazoline was performed in two stages. 4-Chlorobenzaldehyde (**1**), cyclohexane-1,3-dione (**2**), 5-aminotetrazole (**3**), and ethyl chloroacetate (**5**) were used as starting materials, with Whatman filter paper being used as a filter. The synthesis route is depicted in Figure 1.

### 2.2. General Method for Synthesis of Compound 9-(4-Chlorophenyl)-5,6,7,9-tetrahydrotetrazolo[5,1-b]quinazolin-8(4H)-one (4)

The A mixture of compound **1** (2 mmol; 0.283 g), compound **2** (2 mmol; 0.225 g), compound **3** (2 mmol; 0.210 g), and p-TSA (20 mol%; 0.078 g) was heated at 80°C. The progress of reaction was monitored by TLC using a mixture of DCM and MeOH (9 : 1) as mobile phase. After the reaction was completed (10 minutes), the mixture of product was allowed to room temperature and the precipitate was filtered and washed by distilled water. The crude product was allowed to dry in room temperature and then was recrystallized in methanol to afford pure product, compound **4**. The purity of compound **4** was analyzed by HPLC, and the structure was confirmed by spectroscopic analyses, including UV, FTIR, ^1^H-NMR, and HRMS.

Compound **4** was obtained as white solid in 76.5% yield (0.461 g). Melting point: 218–220°C. TLC analysis: *R*_*f*_ = 0.5 (DCM: MeOH = 95 : 5). HPLC: *t*_*R*_ = 3.94 minutes (gradient elution, detector 300 nm). UV (in MeOH): *λ*_max_ = 300 nm. FTIR: 3216, 3057, 1645, 1618, 1553, 1304, 761. ^1^H-NMR (Agilent 500 MHz, DMSO-d_6_): *δ* (ppm): 11.66 (s, 1H), 7.38 (d, 2H), 7.34 (d, 2H), 6.61 (s, 1H), 2.76−2.64 (m, 2H), 2.34−2.23 (m, 2H), 2.01−1.92 (m, 2H). HRMS (*m/z*) was calculated as C_14_H_13_N_5_OCl [M+H]^+^ = 302.0809 and found at 302.0810.

### 2.3. General Method for Synthesis of Ethyl 2-(9-(4-Chlorophenyl)-8-oxo-5,6,7,8-tetrahydrotetrazolo[5,1-*b*]quinazolin-4(6*H*)-yl)acetate (6)

A mixture of compound **4** (1 mmol; 0.3017 g), compound **5** (1.5 mmol; 0.184 g), and K_2_CO_3_ (2 mmol; 0.276 g) was dissolved in DMF (5 mL) and then stirred using a magnetic stirrer at room temperature (∼30°C). The progress of reaction was monitored by TLC using a mixture of EtOAc and *n*-hexane (6 : 4) as the mobile phase. After the reaction was completed (6 hours), the mixture of product was poured into ice water and the precipitate was filtered and washed with distilled water. The crude product was allowed to dry at room temperature to afford pure product, compound **6**. The purity of compound **6** was analyzed by HPLC, and the structure was confirmed by spectroscopic analyses, including UV, FTIR, ^1^H-NMR, ^13^C-NMR, and HRMS.

Compound **6** was obtained as yellowish white solid in 42.1% yield (0.163 g). Melting point: 153-154°C. TLC analysis: *R*_*f*_ = 0.50 (EtOAc : *n*-hexane = 6 : 4). HPLC: *t*_*R*_ = 4.34 minutes (gradient elution, detector 303 nm). UV (in MeOH): *λ*_max_ = 303 nm. FTIR: 3074, 2988, 1755, 1656, 1631, 1555, 1390, 1200, 751. ^1^H-NMR (Agilent 500 MHz, DMSO-d_6_): *δ* (ppm): 7.41 (d, 2H), 7.39 (d, 2H), 6.73 (s, 1H), 4.99 (d, 2H, *J* = 3.2 Hz), 4.22 (q, 2H, *J* = 7.1 Hz), 2.88 (dt, 1H, *J* = 17.7; 5.1 Hz), 2.68−2.62 (m, 1H), 2.36−2.25 (m, 2H), 2.04−1.98 (m, 1H), 1.93−1.87 (m, 1H), 1.24 (t, 3H, *J* = 7.1 Hz). ^13^C-NMR (Agilent 125 MHz, DMSO-d_6_): *δ* (ppm): 194.07, 168.50, 153.36, 150.34, 139.47, 133.66, 129.62, 129.11, 109.25, 62.28, 56.55, 47.51, 35.97, 24.84, 20.65, 14.46. HRMS (*m/z*) was calculated as C_18_H_19_N_5_O_3_Cl [M+H]^+^ = 388.1176 and found at 388.1172.

### 2.4. Molecular Docking

The molecular structures of the tetrazoloquinazoline ligand (compound **6**) and the positive control (doxorubicin) were sketched using ChemDraw Professional 15.0. The 3D structure was then prepared using the Molecular Operating Environment (MOE) 2022.0902 software package with an MMF94x force field and a gradient of 0.0001. A list of the ligands and positive controls is presented in Table 1.

The protein tyrosine kinase was served as the receptor in this study. The molecular structure of this protein was obtained from the protein database with PDB ID 1T46, which had a resolution of 1.60 Å. The crystal structure of this protein was prepared using DSV 2021 (Biovia) and MOE 2022.0901. The original ligand, a water molecule, was removed from the protein.

The protein molecular structure was prepared using the MOE 2022.0901 software package. CHARMM27 was selected as the force field, and various parameters were employed to prepare the protein, including an RMS gradient of 0.01 kcal/mol/Å. Energy minimization was carried out for the H atoms, alpha carbon, and atomic backbone. Finally, the structure was saved in PDB format to be used as a receptor in the docking process. Molecular docking was also performed using MOE 2022.0901 software package.

### 2.5. Molecular Dynamic

Molecular dynamics (MD) simulations were conducted using complexes of the ligand (compound 6) with tyrosine kinase protein. The protein, with a PDB ID of 1t46 and resolution of 1.6 Å, was sourced from the protein database. NAnoscale Molecular Dynamics (NAMD) software version 2.9 was utilized for these preliminary studies. The CHARMM27 (Chemistry at Harvard Macromolecular Mechanics) force field was chosen for its suitability, and each compound was subjected to MD simulations using this force field. A TIP3P water box with a 2.5 Å water layer in each direction was used to simulate the protein environment [16, 17].

The system was gradually heated from 0 K to 300 K over 100 ps using an NVT ensemble. Subsequent MD simulations were performed in an NPT ensemble with periodic boundary conditions for 150 ns, maintaining temperature and pressure coupling at 1 ps intervals. Coordinates were sampled every 0.1 ps for binding free energy calculations and conformational analysis. After heating and equilibration, a 150 ns production MD run in an NPT ensemble was carried out. The results include a 2D graph illustrating the inherent dynamical stability via root-mean-square deviation (RMSD) [18–21].

### 2.6. Adsorption, Distribution, Metabolism, Excretion (ADME)

To gain a better understanding of the physicochemical and pharmacokinetic properties of the drug candidates, as well as to predict drug similarities, we determined the ADME profile. The ADME profile was calculated using the SwissADME server (http://www.swissadme.ch/index.php).

### 2.7. Validation/Test of MCF-7/HER2 Anti-Breast Cancer Activity

The MCF-7/HER2 cell suspension (180 *μ*L) was placed in 96-well plates and incubated in a 5% CO_2_ incubator at 37°C for 24 h to allow the cells to recover after cell harvest. After the incubation period, the MCF-7/HER2 cells were removed from the incubator, and 100 *μ*L of test solution was added for each test concentration into each well (repeated three times). Media controls (RPMI 1640 media), control cells (MCF cells-7/HER2 + RPMI 1640 media), and positive controls (MCF-7/HER2 cells + doxorubicin) were also prepared. A 96-well plate was incubated for 48 h in a 5% CO2 incubator at 37°C. After a 48-hour incubation period, 10 *μ*L of MTT reagent was added to each well, homogenized using an orbital shaker, and incubated again in a 5% CO_2_ incubator at 37°C for 4 h. Next, 200 *μ*L of DMSO was added to each well to dissolve the formazan crystals. Quantitative results were obtained from absorbance values measured at 450 nm using a microplate reader, which corresponds to the maximum absorption.

## 3. Results

### 3.1. Synthesis of Substituted Tetrazoloquinazoline

The A substituted tetrazoloquinazoline (**6**) was successfully synthesized in two stages. The first stage involved the synthesis of compound 9-(4-chlorophenyl)-5,6,7,9-tetrahydrotetrazolo[5,1-b]quinazolin-8(4*H*)-one (**4**) through three-component reaction. This reaction involved 4-chlorobenzaldehyde (**1**), cyclohexane-1,3-dione (**2**), and 5-aminotetrazole (**3**) in the presence of *p*-toluenesulfonic acid (p-TSA) under solvent-free condition (solvent-free method). The second stage is synthesis of ethyl 2-(9-(4-chlorophenyl)-8-oxo-5,6,7,8-tetrahydrotetrazolo[5,1-*b*]quinazolin-4(6*H*)-yl)acetate (**6**) through a nucleophilic substitution reaction between compound **4** and ethyl chloroacetate (**5**) in the presence of potassium carbonate (K_2_CO_3_) in dimethylformamide (DMF). This reaction is depicted in Figure 1.

**Figure 2:**
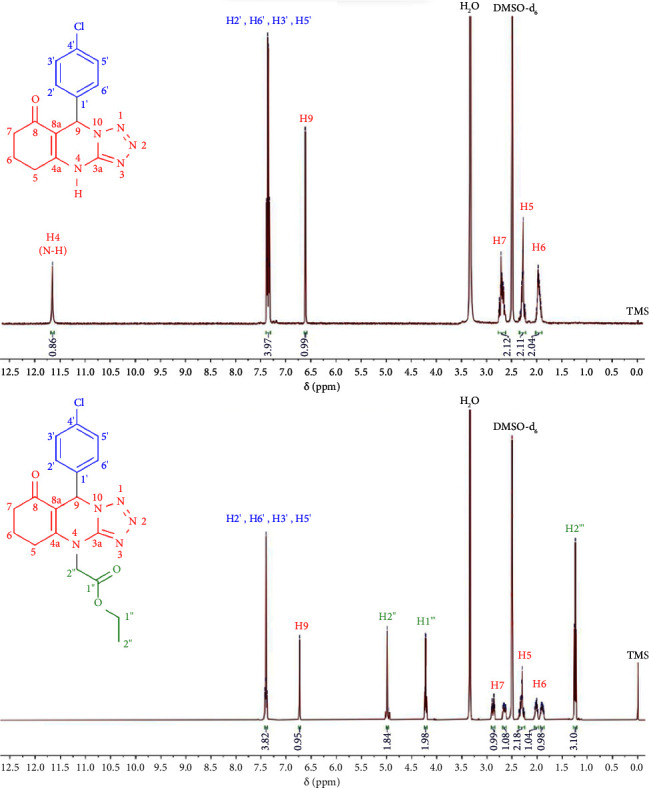
Overlay of ^1^H-NMR spectra of compounds **4** and **6**. The ^1^H-NMR spectra were measured in DMSO-d6 using Agilent 500 MHz.

The results of the ^1^H-NMR analysis of both compounds **4** and **6** confirmed the corresponding number of protons in the synthesized molecules and the target molecules, as presented in Figure 2 and Table 2. In this case, compound **4** exhibited twelve protons; meanwhile, compound **6** has eighteen protons. The ^1^H-NMR spectrum of compound **4** displayed a characteristic signal at 11.66 ppm (s, 1H). The appearance of this signal indicates the presence of a N-H proton (H4) in compound **4**. This signal was no longer observed in the ^1^H-NMR spectrum of compound **6** as the N-H proton has been replaced by an ethyl ester group.

The results of the ^13^C-NMR analysis of compound **6** also showed the suitability of the number of carbons between the synthesized molecules and the target molecules, as can be seen in Table 2. In this case, compound 6 has eighteen carbons. The appearance of signal at 194.07 ppm indicates the presence of carbonyl of ketone (C8); meanwhile, the signal at 168.50 ppm indicates the presence of carbonyl of ester group (C1″). In addition, the appearance of two signals at 62.28 and 14.46 ppm indicates the presence of an ethyl in ethyl ester group of compound **6**. Furthermore, the presence of quinazoline ring in compound **6** was indicated by the appearance of another carbon signals from carbons. Then, the presence of *p*-fluorophenyl ring in compound **6** was indicated by the appearance of two carbon signals at 139.47 (C1′) and 133.66 (C4′) ppm, and two carbon signals at 129.62 (C2″/C6′) and 129.11 ppm (C3″/C5′), with a peak height twice as high as the other peaks. The expansion of the ^13^C-NMR spectra compound **6** is available in Supplementary Materials (Figures SM1–SM12).

### 3.2. Molecular Docking

**Figure 3:**
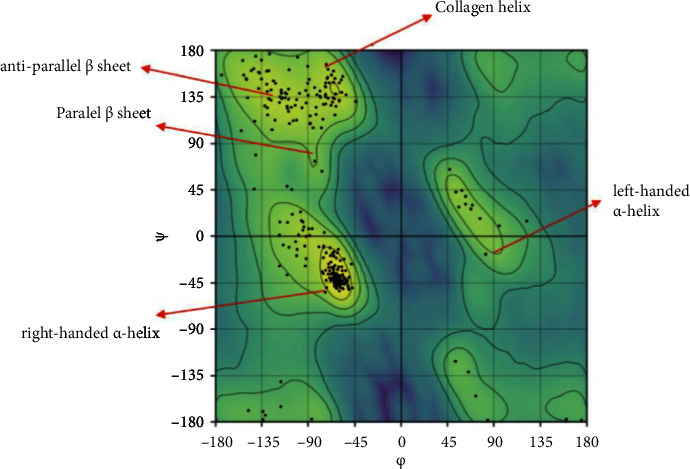
Ramachandran plot for proteins 1 to 46.

The Ramachandran plot is an essential tool in protein structure analysis, aiding the validation of protein conformations and providing insights into their structural characteristics [22, 23]. In this study, the Ramachandran plot was generated online using the https://mohit254-rc-plot-streamlit-app-gsua5l.streamlit.app/website.  Figure 3 presents the Ramachandran plot, indicating that the most favored region is shown in light green, whereas the allowed but rare regions are shown in light green for the alpha helices and beta sheets. Regions in purple are not possible because of steric collisions.

The docking results including ligand positions, root mean square deviation (RMSD) values, and binding free energy values in kcal/mol are presented in Table 3. In this study, the pose with the smallest RMSD value was used to analyze the docking results, as an RMSD <2 is considered valid [24]. The RMSD value indicates as any deviations or errors that may have occurred during the docking process with smaller values indicating smaller deviations or errors.

Another parameter that was considered was the presence of hydrogen bonds. Noncovalent interactions, which help stabilize the structure of macromolecules in cells, are commonly observed in the molecular interactions within the body. These interactions involve the sharing of two electrons between two atoms. Hydrogen bonding specially occurs between positively charged hydrogen atoms and electronegative atoms, such as fluoride (F), nitrogen (N), and oxygen (O) [24]. Additionally, hydrogen bonds can form between the H atom and phenyl ring in a drug compound [25, 26]. Other parameters used to assess the stability of the ligand toward the receptor include hydrophobic and van der Waals bonds. These parameters serve as supporting factors for determining the potential of a compound as a disease inhibitor.

**Figure 4:**
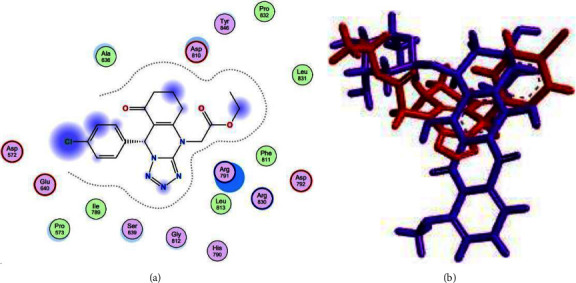
(a) Spatial arrangement of compound **6** and (b) superimposition of compound **6** (red) with positive control (purple).

The visualization results indicated that compound **6** did not form hydrogen bonds because of interactions between proteins and ligands. Instead, compound **6** formed hydrophobic bonds with amino acid residues Arg791 and Arg830. Additionally, compound **6** interacted with the amino acid residues Asp810, Asp792, Glu640, and Asp572 through van der Waals bonds, similar to the positive control (doxorubicin). Considering the docking visualization and the results of the superimposition visualization in Figure 4, compound **6** was classified as inactive with limited anticancer potential.

### 3.3. Molecular Dynamic

MD simulations were performed on protein-ligand complex (i.e., doxorubicin and compound **6**). Based on MD simulations, compound **6** interacts with amino acid residues Asp810, Asp792, Glu640, and Asp572 through van der Waals interactions before and after the simulation. Unfortunately, no hydrogen bonds were established in this compound.

**Figure 5:**
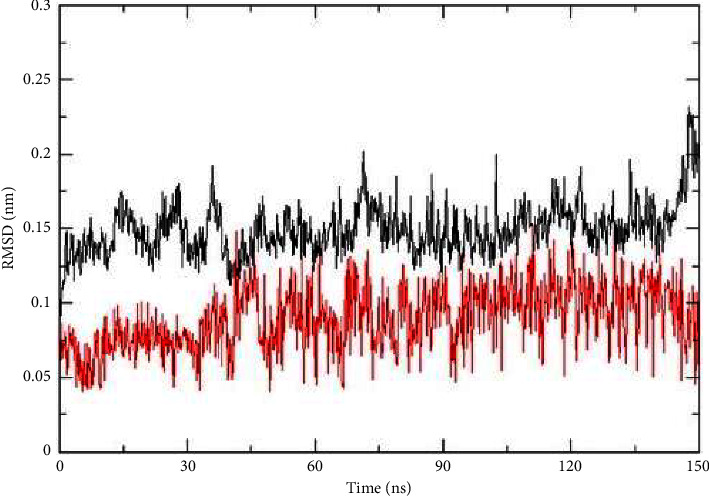
RMSD doxorubicin (red) and compound **6** (black).

Root-mean-square deviation (RMSD) was used to assess the dynamic behavior of the complex. The conformational stability of a complex, which is a structural and dynamic metric, was evaluated using RMSD. A higher RMSD value indicates lower protein stability. According to this analysis, the compound 6-protein complex showed oscillations at 150 ns, with an average RMSD of 0.2 nm. Prior to 40 ns, the compound **6**-protein complex displayed some variation in the average RMSD, but it remained consistent for the duration of the simulation. This complex also had an average RMSD of approximately 0.2 nm. This complex demonstrated stability and strong bonding, as indicated by its low average RMSD values and low volatility, which were almost identical. A lower RMSD value corresponded to greater compound stability.  Figure 5 shows the RMSD of compound **6**.

### 3.4. Adsorption, Distribution, Metabolism, Excretion (ADME)

In this study, the physicochemical properties and ADME were predicted. Physicochemical properties were predicted using SwissADME, which is based on Lipinski's five rules and requires several physicochemical parameters. The advantage of SwissADME is that it presents the predicted results of multiple compounds in a way that facilitates analysis. It displays Boiled-EGG graph that visually represents the simple prediction of compound absorption ability, including the ability to penetrate the blood-brain barrier (BBB). The results of the physicochemical property prediction are presented in Table 4.

### 3.5. Biological Activity

**Figure 6:**
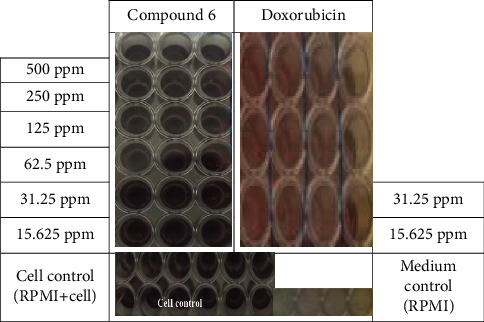
Results of the MTT assay.

The compound resulting from the synthesis of ethyl 2-(9-(4-chlorophenyl)-8-oxo-5,7,8,9-tetrahydrotetrazolo[5,1-b]quinazolin-4(6h)-yl)acetate (compound 6) was tested for cytotoxicity in MCF-7/HER2 breast cancer cells [26, 27]. Cytotoxicity testing was performed using an MTT assay to measure cell viability based on metabolic activity. At a concentration of 250 *μ*g/mL, the color intensity faded compared to that at concentrations of 125 and 62.5 *μ*g/mL. At these lower concentrations, a purple color intensity (fading) was observed compared with the cell and media controls, as shown in Figure 6. The purple color intensity observed in the MTT assay indicated the presence of many live cells capable of reducing the MTT tetrazolium salt (indicating fewer dead cells). Thus, it can be inferred that a significant number of cells died at 250 *μ*g/ml. Subsequently, 100 *μ*L DMSO was added to dissolve the formazan crystals. This step was performed to halt the reaction, lyse the cell membranes, and dissolve formazan crystals that are insoluble in the culture media.

**Figure 7:**
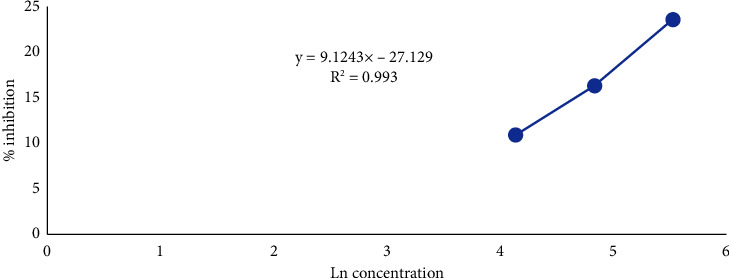
Graph depicting the relationship between the test concentrations and the percentage inhibition values of compound **6**.

The results of the cytotoxicity test were used to calculate the IC_50_, which represents an inhibition concentration of 50. This concentration inhibited the cell growth by 50% in the tested cell population. The IC_50_ value was determined by examining the relationship between the logarithm of the concentration and percentage of cell inhibition caused by compound 6 against MCF-7/HER2 breast cancer cells, as shown in Figure 7. The graph provides a regression equation, typically in the form of *y* = *a* ± *bx* [16].

**Figure 8:**
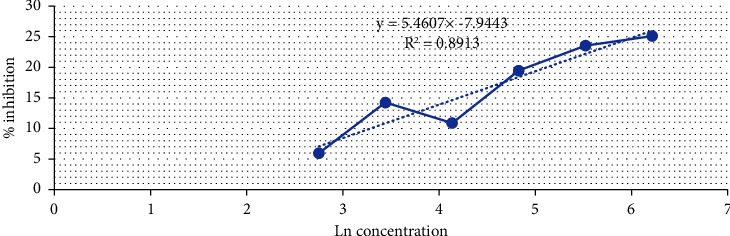
Graph depicting the relationship between the test concentrations and the percentage inhibition values of compound **6** with six concentrations.

Doxorubicin is highly cytotoxic and inhibits topoisomerase II, DNA interactions, cell membrane binding, and the formation of free radicals and oxygen free radicals [28, 29]. According to the French National Cancer Institute (2009), a compound is classified as highly cytotoxic with an IC_50_ value of ≤20 *μ*g/mL, moderately cytotoxic with 21–200 *μ*g/mL, weakly cytotoxic with 201–500 *μ*g/mL, and noncytotoxic with ≥500 *μ*g/mL. Based on this classification, it can be concluded that compound 6 has a low cytotoxic activity against MCF-7/HER2 breast cancer cells.  Table 5 and Figure 8 present the results of the biological activity assay with compound 6. Biological activity for doxorubicin as positive control is depicted in Table 6.

## 4. Discussion

Compound 4 was obtained in very good yield (70–80%), while compound 6 was obtained in acceptable yield (40–50%). The two substituted tetrazoloquinazolines had a sharp melting range (≤2°C) and showed a single spot in their thin-layer chromatography (TLC) profiles (Supplementary Materials, Figure SM1). The HPLC chromatogram also showed a single peak, indicating excellent purity of the two compounds (Supplementary Materials, Figure SM2). The UV spectra of the two compounds appear very similar owing to the similarity in their chromophores. However, there was a slight difference between the *λ* maximum values. Compound 4 had a maximum wavelength of 300 nm, whereas compound 6 had a maximum of 300 nm (Supplementary Materials, Figure SM3). The conversion of 4 to 6 could be clearly monitored through the overlay of the FTIR spectra of compounds 4 and 6 (Supplementary Materials, Figure SM4). The absorption band of the N-H group (H4) appeared only in the FT-IR spectrum of compound 4 (highlighted in black). Meanwhile, the absorption band of the C=O ester appeared only in the FTIR spectrum of compound 6 (highlighted in purple). This result shows that the introduction of an ethyl ester substituent was successful.

The presence of ethyl ester group in compound **6** was also supported by the appearance of three new signals in the aliphatic proton area at 1.24 ppm (t, 3H), 4.23 ppm (q, 2H), and 4.99 (d, 2H) (highlighted in green). In addition, the presence of quinazoline ring in compound **4** was indicated by the appearance of four signals from protons H9 (s, 1H), H7 (m, 2H), H5 (m, 2H), and H6 (m, 2H) in the range of 6.61−1.92 ppm. Meanwhile, in the compound **6**, the quinazoline protons appeared as six signals, H9 (s, 1H), H7 (dt, 1H) and (m, 1H), H5 (m, 2H), and H6 (m, 1H) and (m, 1H) in the range of 6.73−1.87 ppm. Then, the presence of *p*-fluorophenyl ring in both compounds **4** and **6** was indicated by the appearance of two roofing doublets around 7.42−7.38 ppm (d, 2H) and 7.39−7.33 ppm (d, 2H). The expansion of the ^1^H-NMR spectra of both compounds **4** and **6** can be seen in Supplementary Materials (Figures SM8 and SM9).

The mass analysis was performed to ensure the molecular weight of both compounds **4** and **6**. The molecular ion mass of compound **4** was calculated as C_14_H_13_N_5_OCl [M+H]^+^ = 302,0809, and the molecular ion peak was found at 302.0810 in the HRMS spectrum of compound **4**. Furthermore, the molecular ion mass of compound **6** was calculated as C_18_H_19_N_5_O_3_Cl [M+H]^+^ = 388.1176 and the molecular ion peak was found at 388.1172 in the HRMS spectrum of compound **6**. There is only a very small difference between the calculated and found mass (0.0001–0.0004). In addition, the HRMS spectra of both compounds 4 and 6 were also showed the isomer peaks of ^35^Cl and ^37^Cl with the peak high ratio of 3 : 1, as can be seen in Supplementary Materials (Figures SM11 and SM12). Overall, the result of spectroscopic analyses showed a structural match between the obtained molecule and the target molecule.

According to the Protein Data Bank (https://www.rcsb.org/structure/1t46), no Ramachandran outliers have been reported for the protein backbone or sidechain. Thus, the protein structure was validated for use as a protein target.

**Figure 9:**
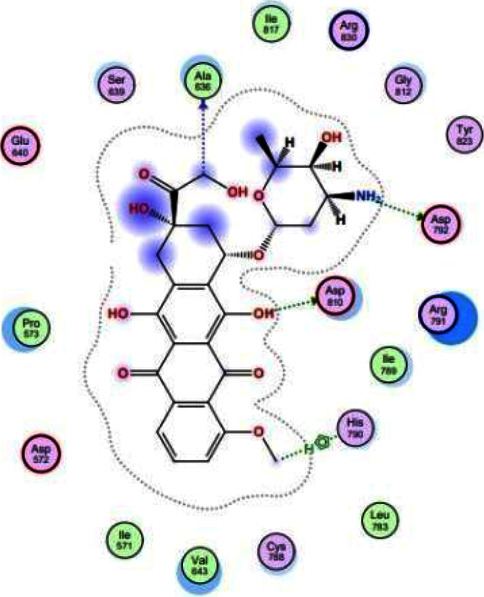
Spatial arrangement of doxorubicin as positive control.

Based on the docking results, the positive control (doxorubicin) had a bond-free energy value of −8.7719 kcal/mol with an RMSD value of 1.0645. Table 3 shows that the positive control (doxorubicin) was able to bind to 18 amino acid residues on the active site of the receptor. These amino acids were Asp792, Asp810, His790, Ala636, Arg830, Arg791, Glu640, Asp572, Ser639, Ile817, Gly812, Tyr823, Ile789, Leu783, Cys788, Val643, Ile571, and Pro573. The visualization results indicated that doxorubicin can form hydrogen bonds with the amino acid residues Asp792, Asp810, and His790. All three were bonded to their respective hydroxyl groups via hydrogen bonding. The hydroxy group that acts as a hydrogen bond donor is marked with a green dotted line, with an arrow pointing to amino acids Asp792 and Asp810. The hydroxy group that acts as a hydrogen bond acceptor is marked with a green dotted line with an arrow pointing toward the hydroxy group. The van der Waals bond formed between the Glu640 and Asp572 amino acid residues is marked with a red ring. In addition, doxorubicin has an active site on the acidic residues of the Ala636, Arg830, and Arg791 proteins, with hydrophobic bonds marked with a blue ring. The spatial arrangement of doxorubicin is shown in Figure 9.

Based on the docking results for the quinazoline derivatives, the calculated bond-free energy value was −6.9970 kcal/mol, and the RMSD value of 0.7798, respectively. Compound 6 shares 12 amino acids with the positive control, especially Arg791, Arg830, Asp810, Asp792, Glu640, Asp572, Ala636, His790, Gly812, Ser639, Ile789, and Pro573.

Compound **6** was subjected to molecular dynamics (MD) simulations to examine interactions between ligands and receptors [17]. The stability of the MD simulations was evaluated to confirm the binding profile of the ligands and estimate their activity. The simulation was run for 150 ns [18–21] to ensure the maintenance of the connection between the protein and active compounds in the current study. To assess the affinity of the ligand for the binding site, the simulation began with a high stability at an energy minimum of 300 K.

The another parameter for the MD simulation is RMSD. RMSD has traditionally been used to measure the separation between atomic chains of proteins, assessing differences in two protein structures over simulation intervals. A higher RMSD value indicates greater dissimilarity, whereas a value of zero indicates an identical conformational structure. As shown in Figure 5, compound **6** was stable as their RMSD values were not very high.

Drug-likeness or drug similarity is a method used to assess the likelihood of a molecule being an oral drug by considering the bioavailability of the compound. The SwissADME web-based software offers an analysis of drug-likeness that provides access to five different types of rules, each with its own range of drug-like categories. In this study, we conducted a drug-likeness analysis based on the Lipinski rule, also known as the Rule of Five (Ro5). The parameters of Lipinski's rule include Log P, molecular weight, hydrogen bond acceptors (HBA), and hydrogen bond donors (HBD). To obtain the SMILES code for the SwissADME application, we used MarvinSketch to depict the chemical structure.

According to Lipinski et al. [13], a compound will have low permeability and is difficult to absorb if it has a molecular weight greater than 500, a logarithm of the partition coefficient of octanol/water (log P) greater than 5, more than 5 hydrogen bond donors (HBD) represented by the number of O-H and N-H groups, and more than 10 hydrogen bond acceptors (HBA) represented by the number of O and N atoms [30]. Molecular weight directly affects the ability of a compound to pass through cell membranes via passive diffusion. If the molecular weight (MW) of a compound is greater than 500 g/mol, it becomes increasingly difficult for it to diffuse across the membranes.

The log P parameter describes how well a compound dissolves in octanol/water (a biological membrane). A higher log *P* value indicates increased hydrophobicity, indicating that the compound is more likely to be toxic. Hydrophobic compounds are problematic because they persist longer in the lipid bilayer and spread throughout the body, reducing target binding selectivity. On the other hand, if the log *P* value becomes more negative, it becomes more difficult for the compound to cross the lipid bilayer. Parameters such as hydrogen bond donors and acceptors are used to describe the ability of a compound to form hydrogen bonds, which is necessary for the absorption process. Therefore, if a compound has more than five hydrogen bond donors and more than 10 acceptors, it requires more energy to be absorbed.

Hydrogen bonding can affect the chemical and physical properties of a compound, including boiling and melting points, water solubility, chelation ability, and acidity. Lipinski's Rule of Five is used to describe the solubility of compounds that passively diffuse into cells [31, 32].

According to Table 4, quinazoline derivative compounds fulfill Lipinski's Rule of Five parameters without any deviations. This suggests that compound **6** is easily absorbed and has good permeability. If a compound fails to meet Lipinski's Rule of Five, there is a likelihood of absorption-related issues when taken orally as described by Lipinski (2004) [33–35]. However, complying with Lipinski's Rule of Five does not guarantee good activity, as it does consider the specific chemical structures present in a compound.

Based on the results of molecular docking and ADME studies, it can be concluded that compound **6** is similar to the ligand and positive control (doxorubicin). Therefore, quinazoline derivatives can be considered as test compounds that are easily absorbed and possess similar properties.

MTT assay is a sensitive, quantitative, and reliable colorimetric test that measures cell viability, proliferation, and activity. The principle of this method involves the conversion of the yellow tetrazolium salt MTT into a purple formazan compound. MTT is absorbed by living cells, and a reduction reaction occurs through the action of reductase enzymes in the mitochondrial respiratory chain, resulting in the formation of purple formazan. The intensity of the formazan color was correlated with the number of live cells. Therefore, the color intensity increased with the number of live cells [36].

A higher absorbance value indicates a greater number of live cells, as indicated by the purple color. Absorbance was used to calculate the percentage of cell inhibition. Cell inhibition refers to the inability of the cells to survive. A higher percentage of inhibition corresponds to a higher percentage of dead cells. Subsequently, the percentage inhibition was used to calculate IC_50_ values. IC_50_ (inhibition concentration 50) is the concentration that results in a 50% inhibition of cell growth in the cell population under test. A higher IC_50_ value indicated that the compound was less toxic.

Based on the results of the MTT assay, the IC_50_ value of compound 6 was found to be 4689.78 *μ*g/mL (>1000 *μ*g/mL), indicating that the compound was inactive. In contrast, the positive control doxorubicin showed an IC_50_ value of 0.02 *μ*g/mL, indicating that the positive control is active. Doxorubicin belongs to the anthracycline group and is commonly used in breast cancer treatment [37].

## 5. Conclusions

Ethyl 2-(9-(4-chlorophenyl)-8-oxo-5,7,8,9-tetrahydrotetrazolo[5,1-B]-4(6H)-yl) acetate was synthesized based on in silico studies through molecular docking and MD simulation, suggesting that this compound may not have the potential to act as an anticancer inhibitor. The binding free energy (Δ*G*) of the substituted tetrazoloquinazoline (compound **6**) was significantly different from that of the positive control (doxorubicin). However, ADME testing of the quinazoline derivative indicated that it had high permeability and was easily absorbed. Additionally, the MTT assay classified the quinazoline derivative compound as noncytotoxic against breast cancer cells, with an IC_50_ value greater than 1000 *μ*g/mL. This is an early stage for the identification of potential compounds against breast cancer. However, further in vivo evaluations are required to confirm the potency of compound **6** and pave the way for the discovery of promising next-generation drugs against breast cancer.

## Figures and Tables

**Table 1:** Molecular structure of ligands.

Compound	Molecular structure
Compound **6**	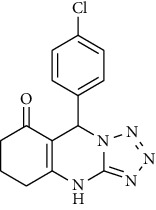

Doxorubicin (positive control)	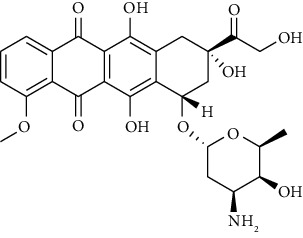

**Table 2:** Interpretation of ^1^H-NMR spectroscopic data of compounds **4** and **6**.

Atomic numbering	Compound **4**	Compound **6**	
	*δ*_*H*_ (ppm)^∗^	*δ*_*H*_ (ppm)^∗^	*δ*_*C*_ (ppm)^∗∗^
3a	—	—	153.36
4	11.66 (s, 1H)	—	—
4a	—	—	150.34
5	2.34−2.23 (m, 2H)	2.36−2.25 (m, 2 H)	25.84
6	2.01−1.92 (m, 2H)	2.04−1.98 (m, 1H), 1.93−1.87 (m, 1H)	20.65
7	2.76–2.64 (m, 2H)	2.88 (dt, 1H, *J* = 17.7; 5.1 Hz) 2.68−2.62 (m, 1H)	35.97
8	—	—	194.07
8a	—	—	109.25
9	6.61 (s, 1H)	6.73 (s, 1H)	56.55
1′	—	—	139.47
2′/6′	7.38 (d, 2H)	7.41 (d, 2H)	129.62
3′/5′	7.34 (d, 2H)	7.39 (d, 2H)	129.11
4′	—	—	133.66
1″	—	—	168.50
2″	—	4.99 (d, 2H, *J* = 3.2 Hz)	47.51
1″	—	4.22 (q, 2H, *J* = 7.1 Hz)	62.28
2″	—	1.24 (t, 3H, *J* = 7.1 Hz)	14.46

^∗^The ^1^H-NMR spectra were measured using Agilent 500 MHz. ^∗∗^The ^13^C-NMR spectrum was measured using Agilent 125 MHz.

**Table 3:** Docking results.

Compound	*S* (kcal/mol)	RMSD	H bond	Hidrofobik	van der Waals	The others interaction	Binding factor
Doxorubicin (positive control)	−8.7719	1.0645	**Asp792, Asp810, His790,**	**Ala636, Arg830, Arg791**	**Glu640, Asp572**	**Ser639, Ile817, Gly812, Tyr823, Ile789, Leu783, Cys788, Val643, Ile571, Pro573**	18
Compound **6**	−6.9970	0.7798	—	**Arg791, Arg830**	**Asp810, Asp792, Glu640, Asp572**	**Ala636,** Tyr864, Pro832, Leu831, Phe811, Leu813, **His790, Gly812, Ser639, Ile789, Pro573**	12

The bold values indicate amino acids that bind between compound 6 and the protein are the same as those that bind between the positive control and the protein.

**Table 4:** Results for SwissADME.

No	Molecular weight (g/mol)	Log of the partition octanol and water	Hydrogen bond donor (HBD)	Hydrogen bond acceptor (HBA)	Rotatable bond	Drug-likeness
Compound **6**	387.82	2.41	0	6	5	Yes
Positive control (doxorubicin)	543.52	0.52	6	12	5	No
Parameter *Rule of Five*	≤500	≤5	≤5	≤10	—	Yes

**Table 5:** Inhibition concentration for compound **6**.

Concentration (*µ*g/mL)	Ln concentration	% inhibition	IC_50_ (*µ*g/mL)
500 250	6.214 5.521	25.114 23.55	>1000
125	4.828	16.31	
62.5 31.25 15.625	4.135 3.442 2.748	10.90 14.237 5.927	

**Table 6:** Inhibition concentration for doxorubicin.

Concentration (*µ*g/mL)	Ln concentration	Absorbance				% inhibition	IC_50_ (*µ*g/mL)
		1	2	3	Average		
250	5.521	0.231	0.178	0.161	0.190	85.24	0.023
125	4.828	0.304	0.204	0.189	0.232	81.94	
62.5	4.135	0.317	0.221	0.222	0.253	80.01	

## Data Availability

The data utilized and/or examined in this study were included in this article and further details are available from the corresponding author.

## Abbreviations

ADME: Adsorption, distribution, metabolism, excretion
ER: Estrogen receptor
FTIR: Fourier-transform infrared spectroscopy
1H-NMR: Hydrogen-1 nuclear magnetic resonance
HER2: Human epithelial receptor 2
MCF7: Michigan cancer foundation-7
MD: Molecular dynamic
MTT: 3-[4,5-Dimethylthiazol-2-yl]-2,5 diphenyl tetrazolium bromide
PR: Progesterone receptor
RMSD: Root-mean-square deviation
Ro5: Rule of Five
TLC: Thin-layer chromatography
WHO: World Health Organization.

## Supplementary Materials

Supplementary Materials Supplementary materials including the TLC profile, HPLC chromatograms, UV spectra, FTIR, ^1^H-NMR, ^13^C-NMR, and HRMS spectrum for compounds **4** and **6**.
