# Supplementary material for: Exploring Substituted Tetrazoloquinazoline: Biological Activities, Molecular Docking Analysis, and Anti-Breast Cancer MCF7/HER2 Effects
Source: Adv Pharmacol Pharm Sci. 2024 Aug 28;2024:6952142. doi: 10.1155/2024/6952142 (PMC11374424; doi:10.1155/2024/6952142)
Supplement: Supplementary Materials — including the TLC profile, HPLC chromatograms, UV spectra, FTIR, 1H-NMR, 13C-NMR, and HRMS spectrum for compounds 4 and 6. [file 6952142.f1.docx]

**SUPPLEMENTARY MATERIALS (SM)**

**Exploring Substituted Tetrazoloquinazoline: Biological Activities, Molecular Docking Analysis, and Anti-Breast Cancer MCF7/HER2 Effects**

Neni Frimayanti^*^, Ihsan Ikhtiarudin, Rahma Dona, Rahul Oktarizal, Aprilia Cindy Nurfatimah

Department of Pharmacy, Sekolah Tinggi Ilmu Farmasi Riau, Jalan Kamboja, Simpang Baru, Pekanbaru, 28293 Indonesia.

Corresponding author: [nenifrimayanti@gmail.com](mailto:nenifrimayanti@gmail.com)


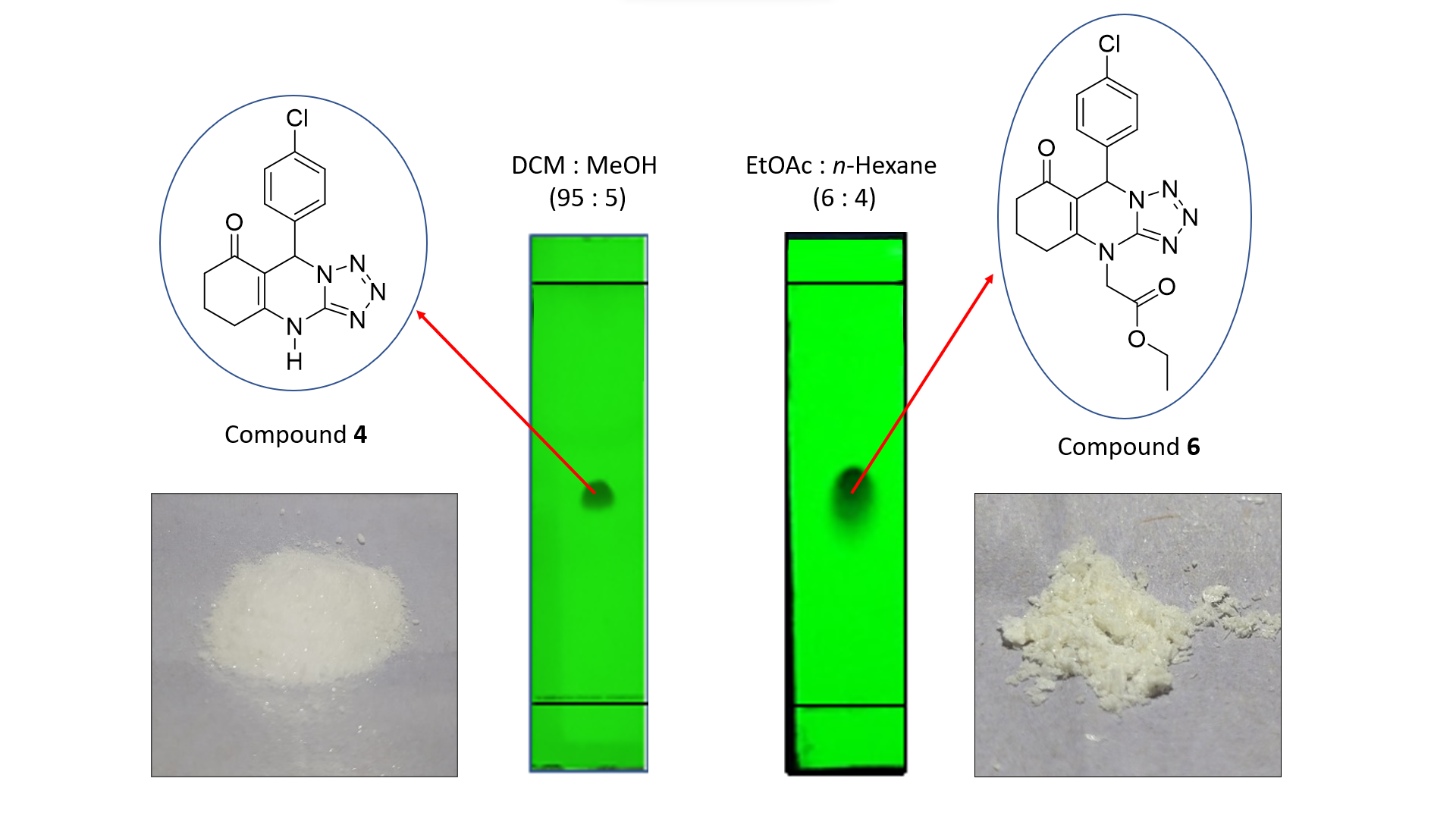


**Figure SM 1**. TLC profiles of compounds **4** and **6** using silica gel F_254_ plates. Compound **4** was analysed using a mixture of dichloromethane (DCM) and methanol (MeOH) as mobile phase. Compound **6** was analysed using a mixture of ethyl acetate (EtOAc) and *n*-hexane as mobile phase.


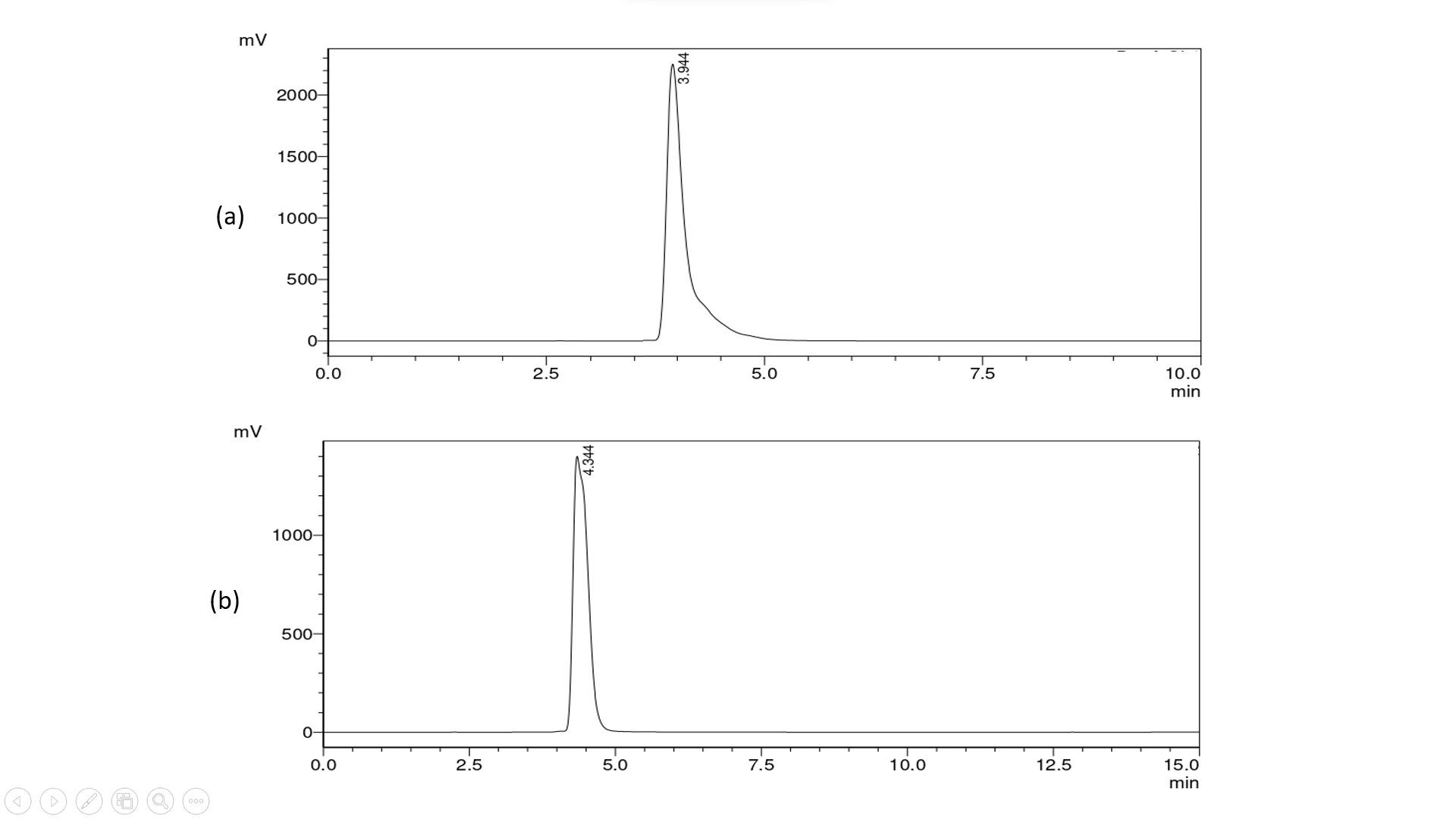


**Figure SM2.** HPLC chromatograms of compounds **4** and **6**, (a) HPLC chromatogram of compound **4**, (b) HPLC chromatogram of compound **6**


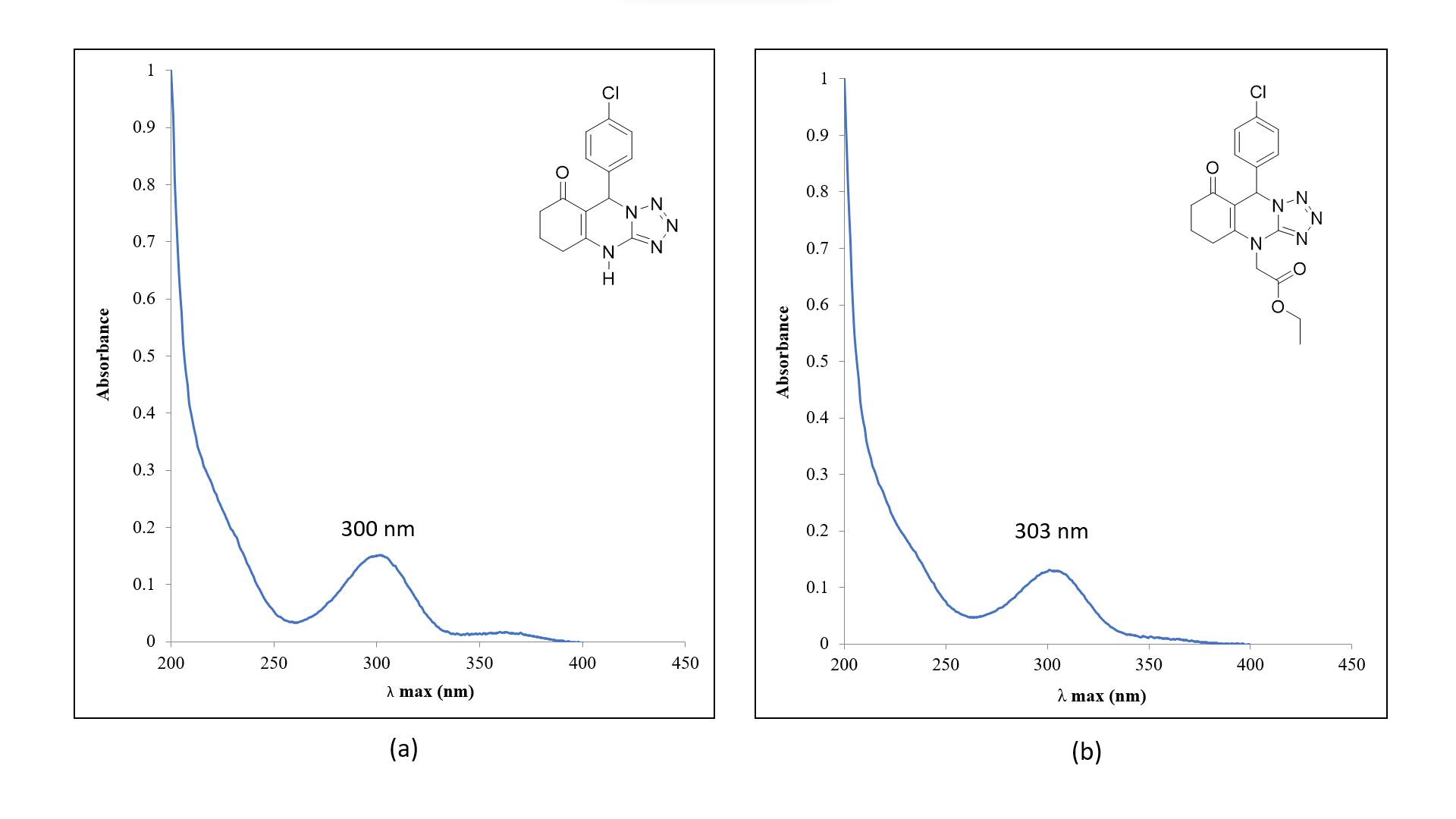


**Figure SM3.** UV spectra of compounds **4** and **6**, (a) UV spectrum of compound **4**, (b) UV spectrum of compound **6**


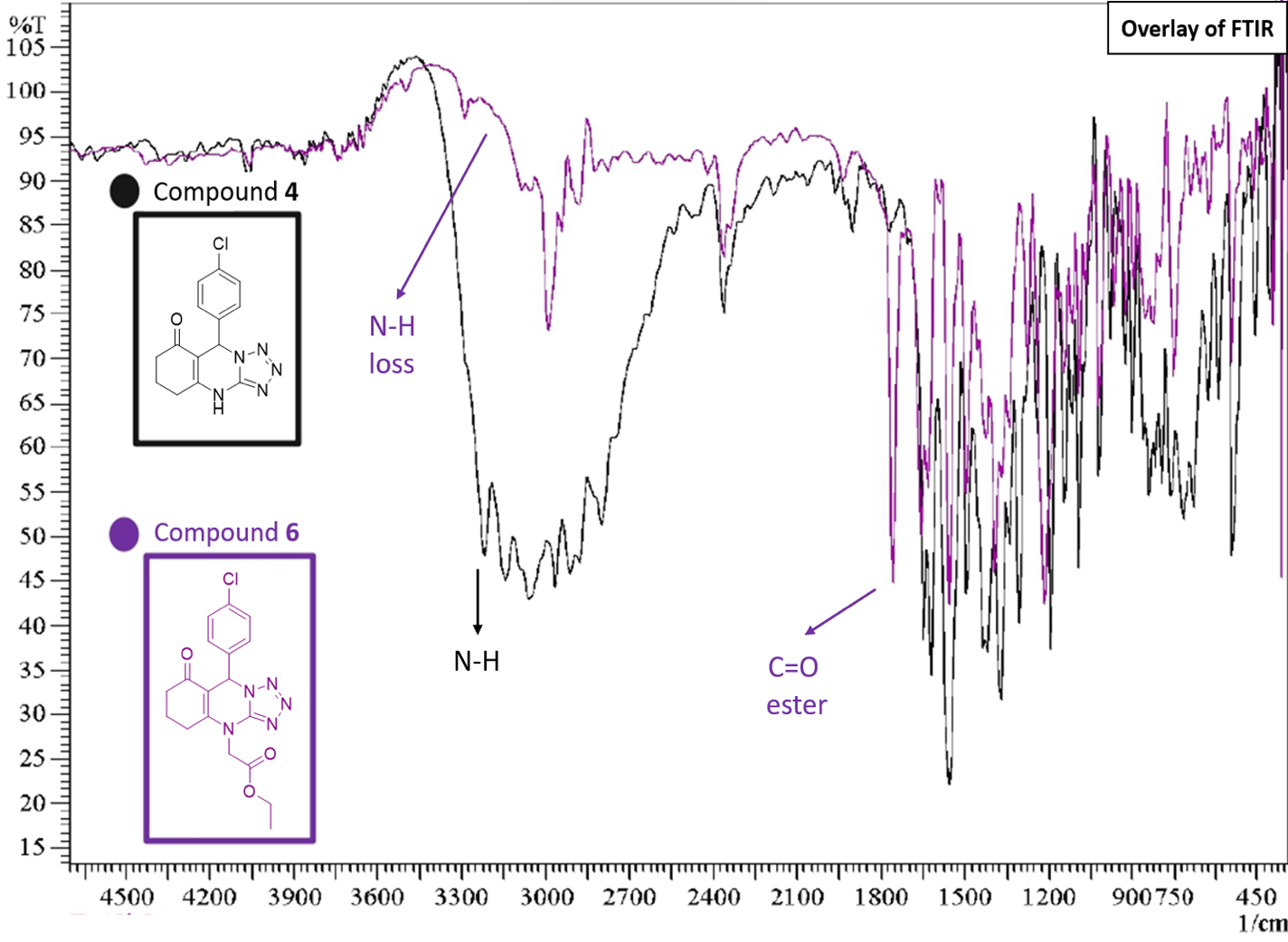


**Figure SM4**. Overlay of FTIR spectra of compounds **4** and **6.** FTIR spectrum of compound **4** was highlighted in black and FTIR spectrum of compound **6** was highlighted in purple


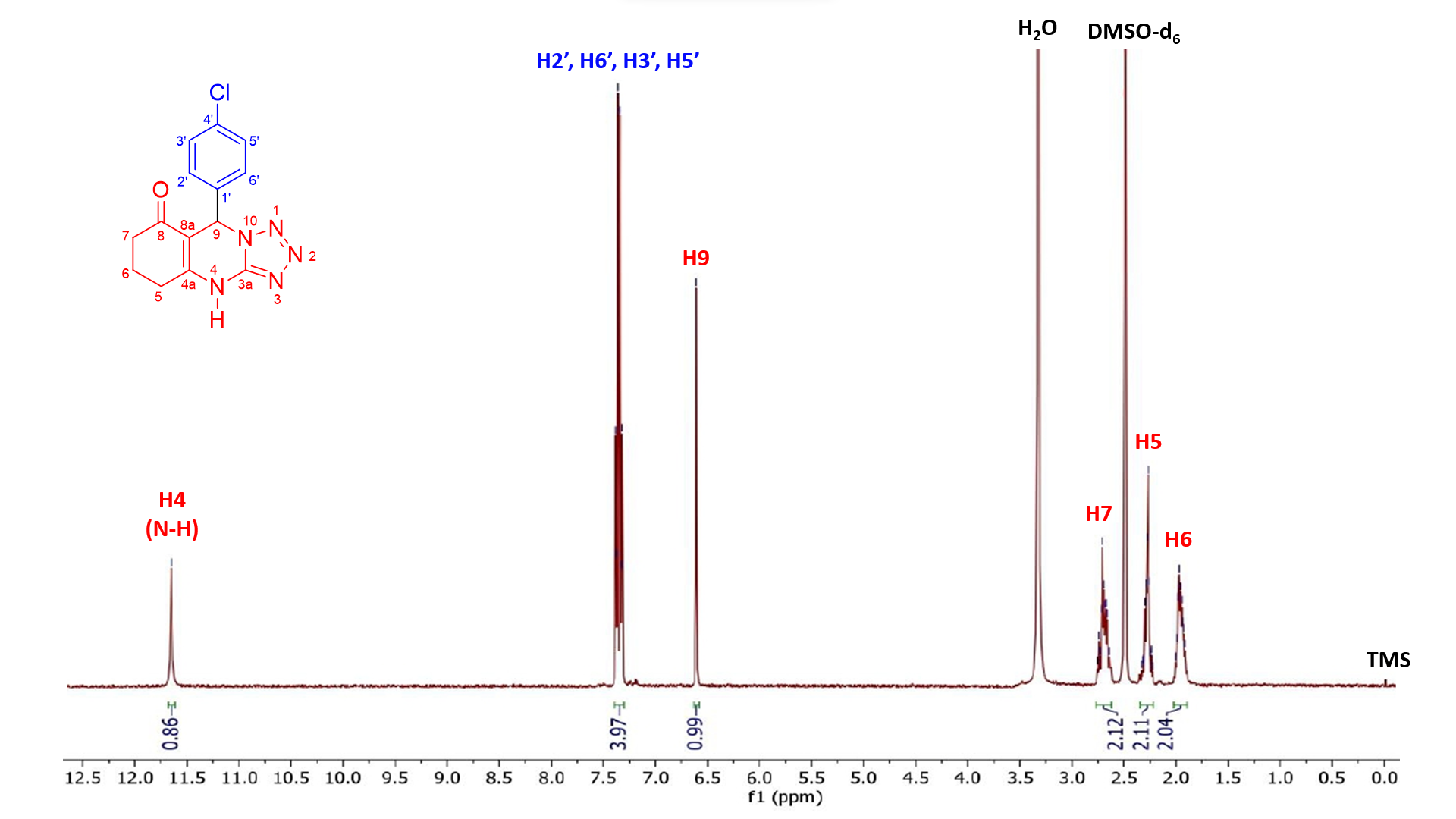


**Figure SM5**. ^1^H-NMR spectrum of compound **4**, measured in DMSO-d6 using Agilent 500 MHz


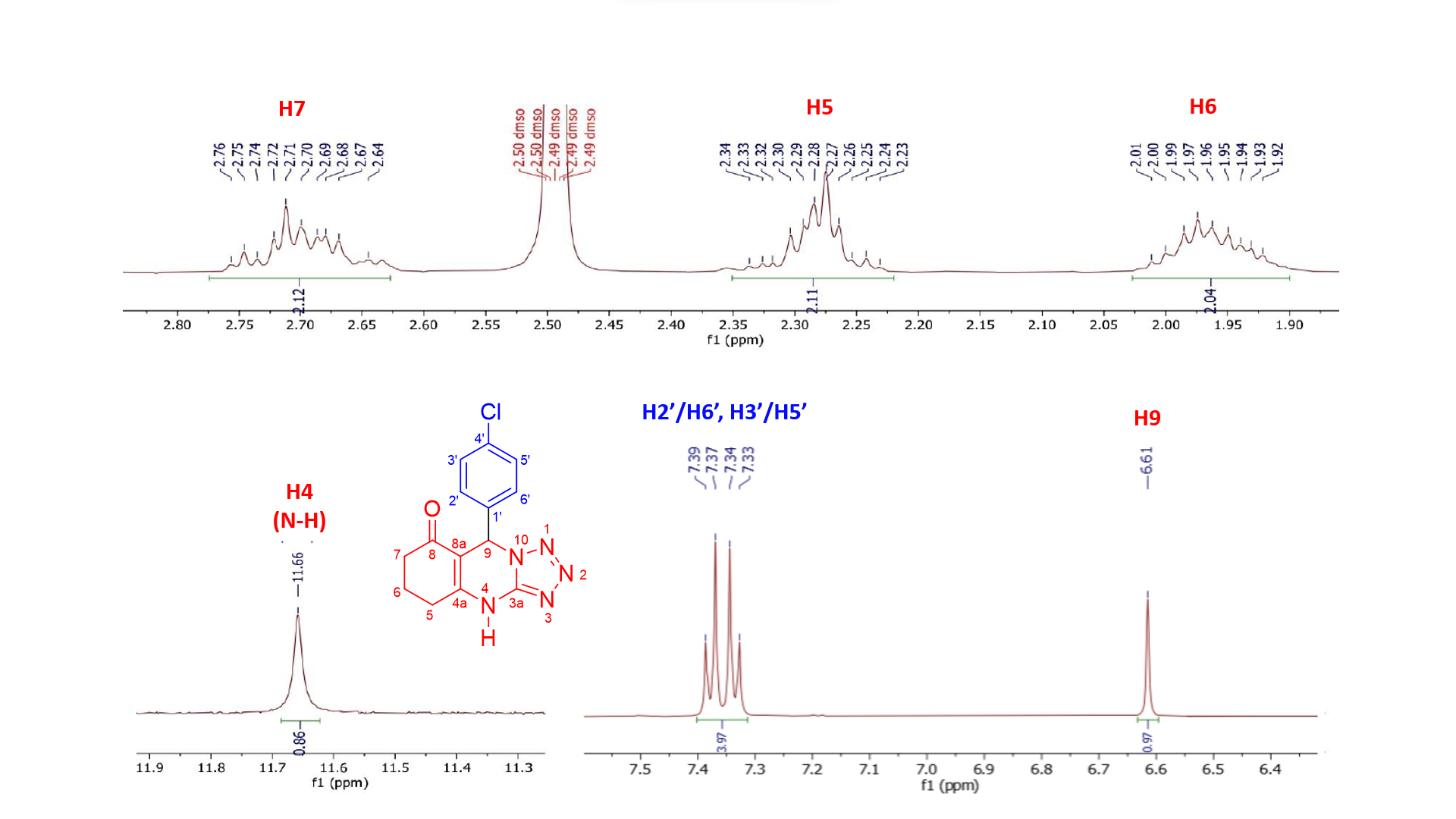


**Figure SM6**. The expansion of ^1^H-NMR spectrum of compound **6**, measured in DMSO-d6 using Agilent 500 MHz


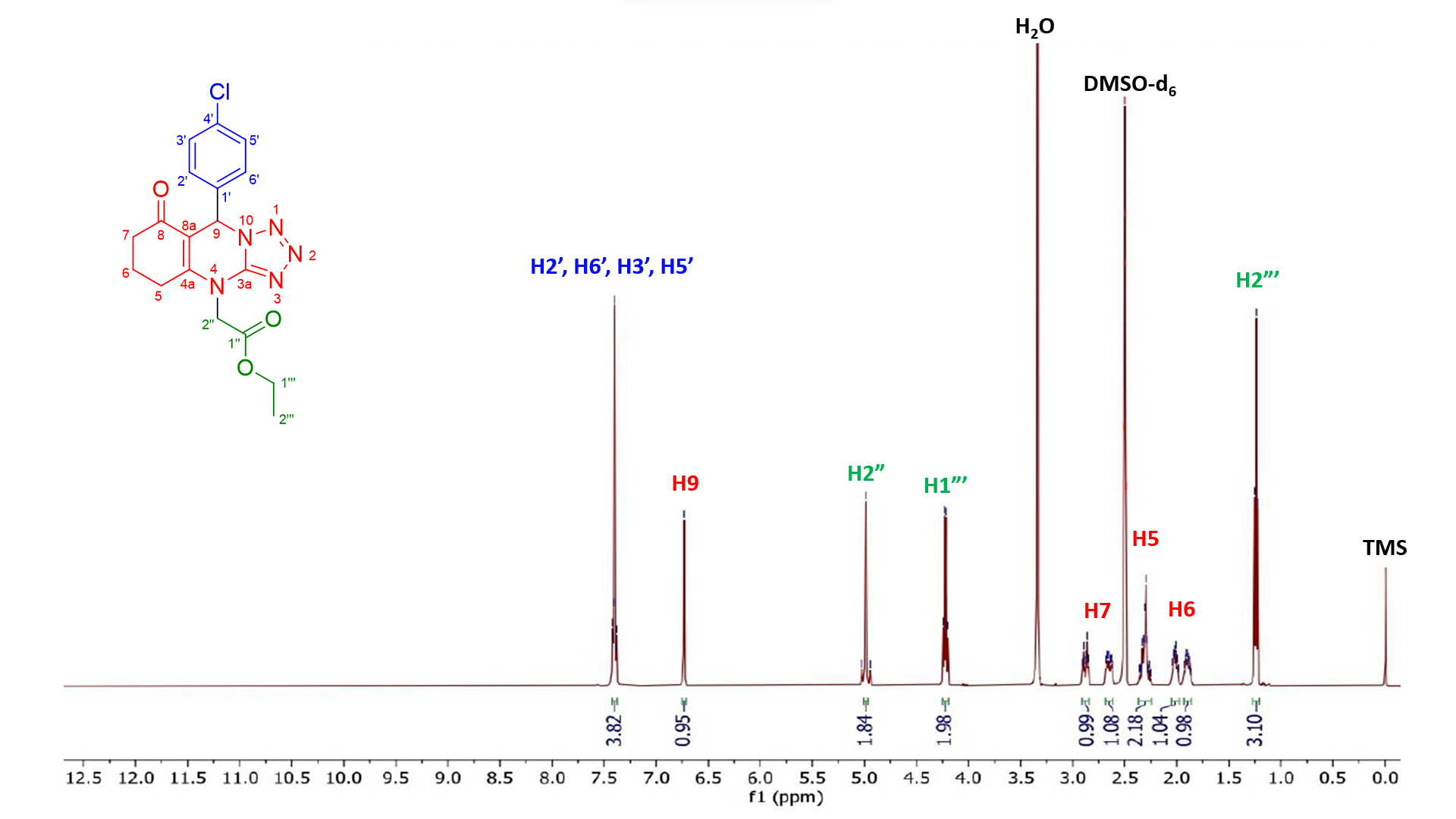


**Figure SM7**. ^1^H-NMR spectrum of compound **6**, measured in DMSO-d6 using Agilent 500 MHz


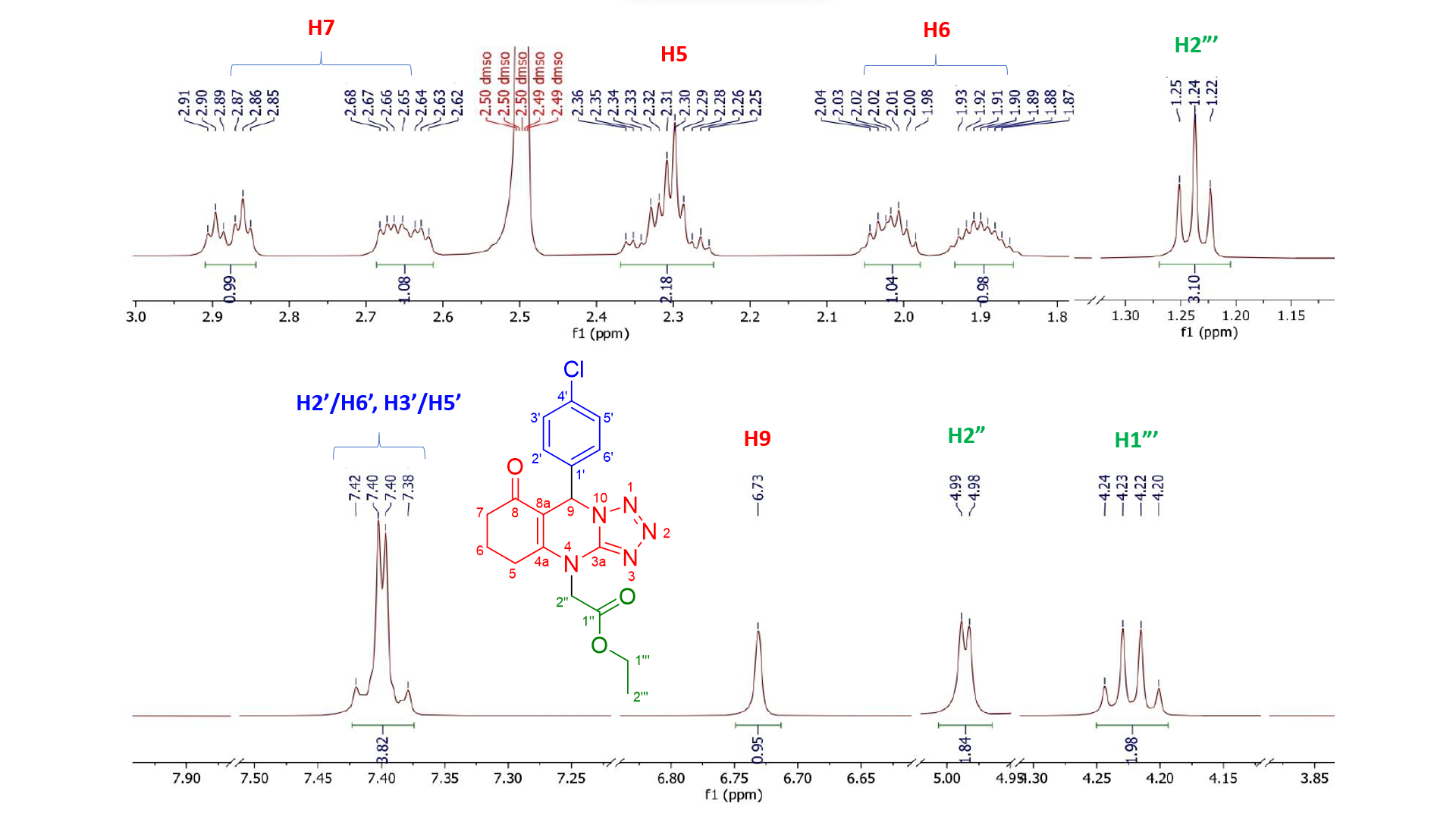


**Figure SM8**. The expansion of ^1^H-NMR spectrum of compound **6**, measured in DMSO-d6 using Agilent 500 MHz


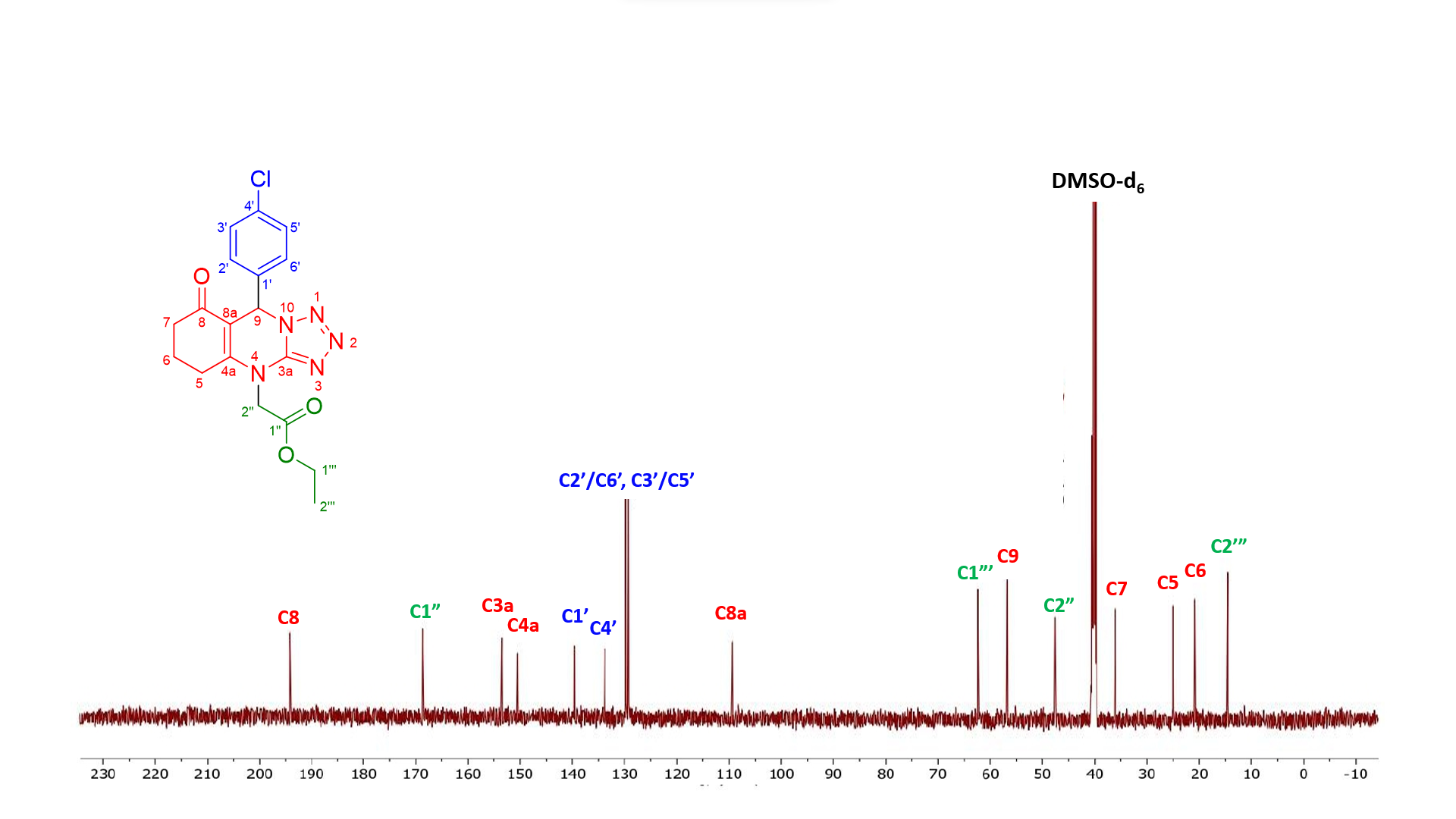


**Figure SM9**. ^13^C-NMR spectrum of compound **6**, measured in DMSO-d6 using Agilent 125 MHz


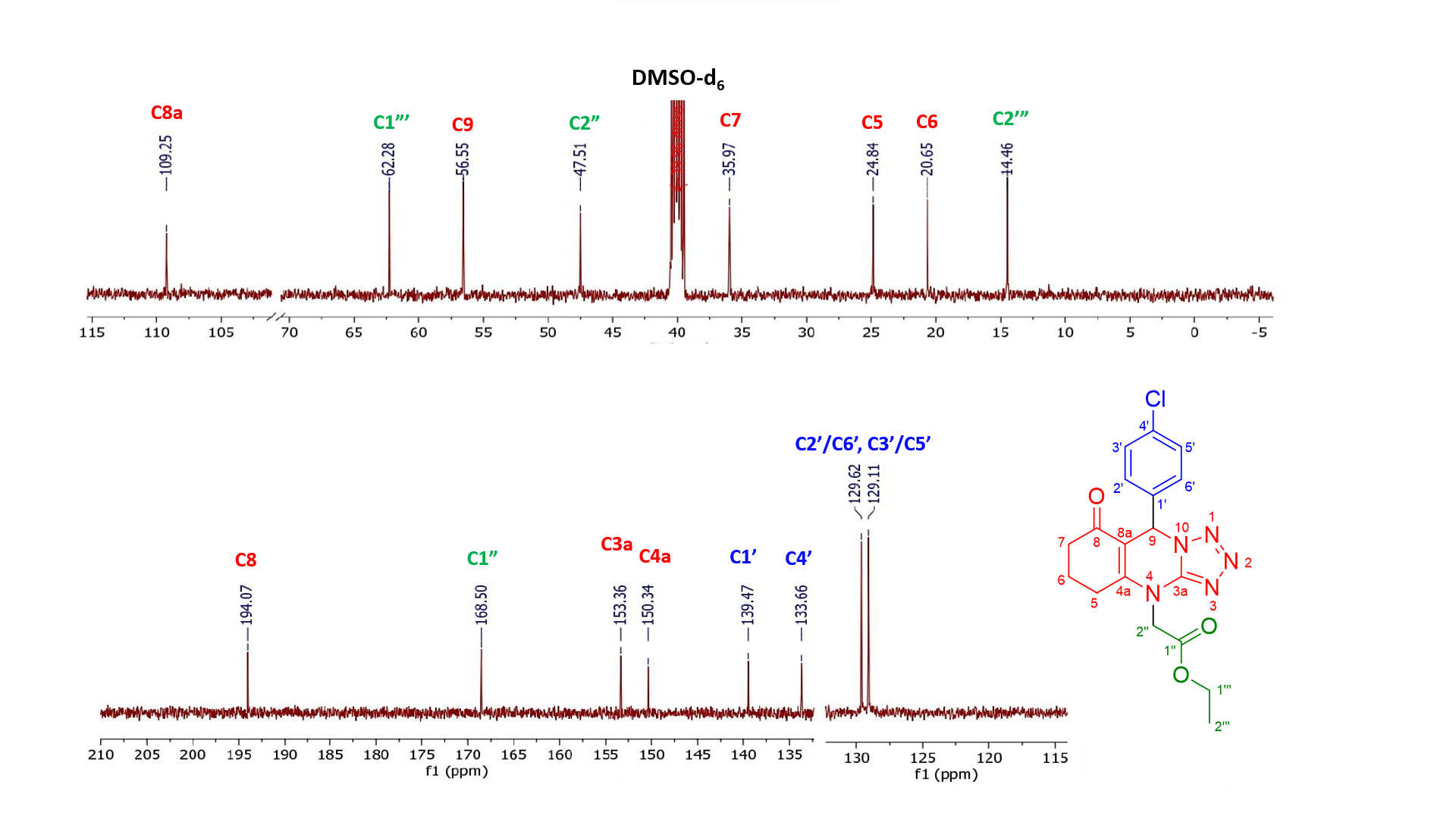


**Figure SM10**. The expansion of ^13^C-NMR spectrum of compound **6**, measured in DMSO-d6 using Agilent 125 MHz


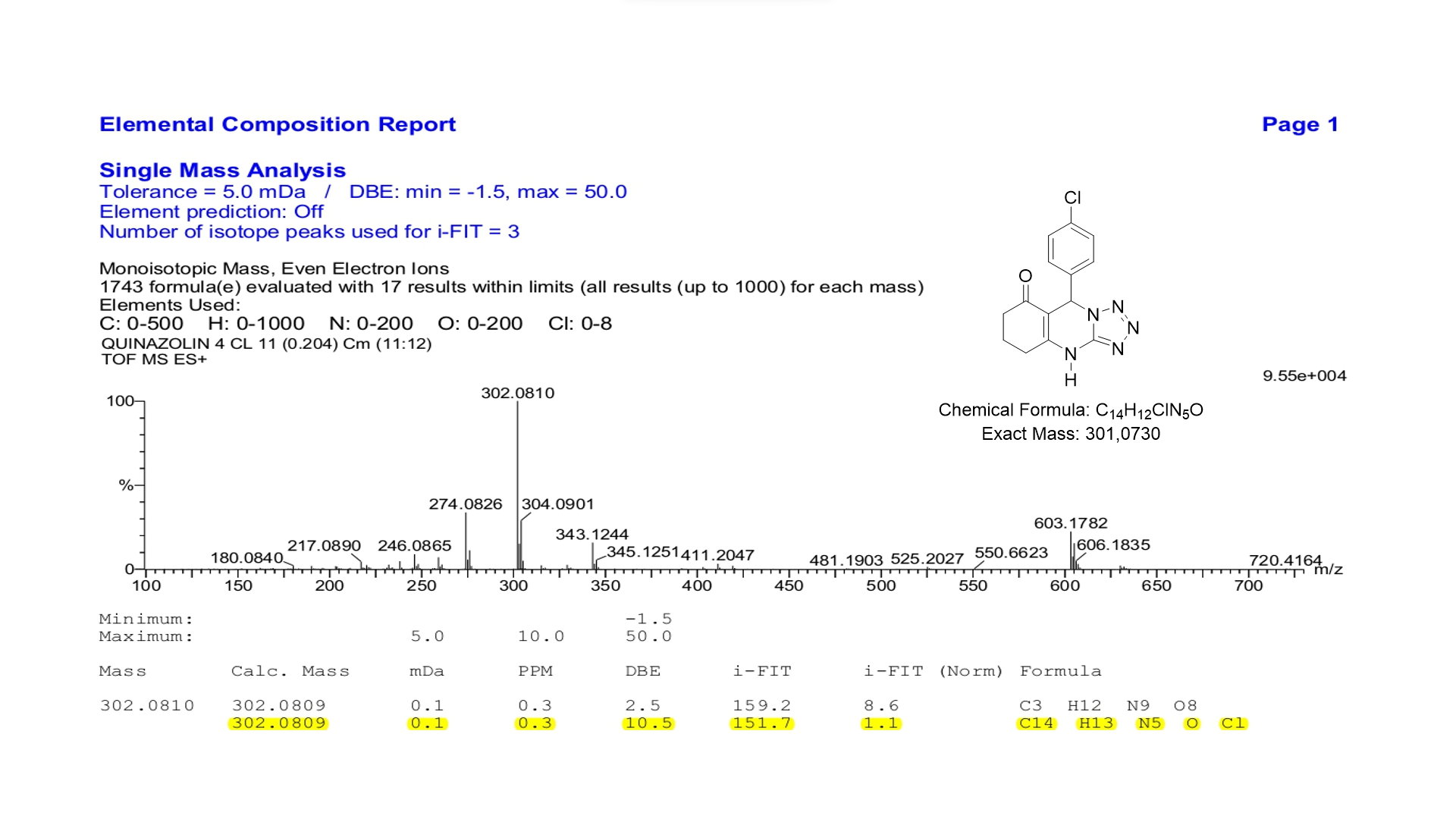


**Figure SM11**. HRMS spectrum of compound **4**


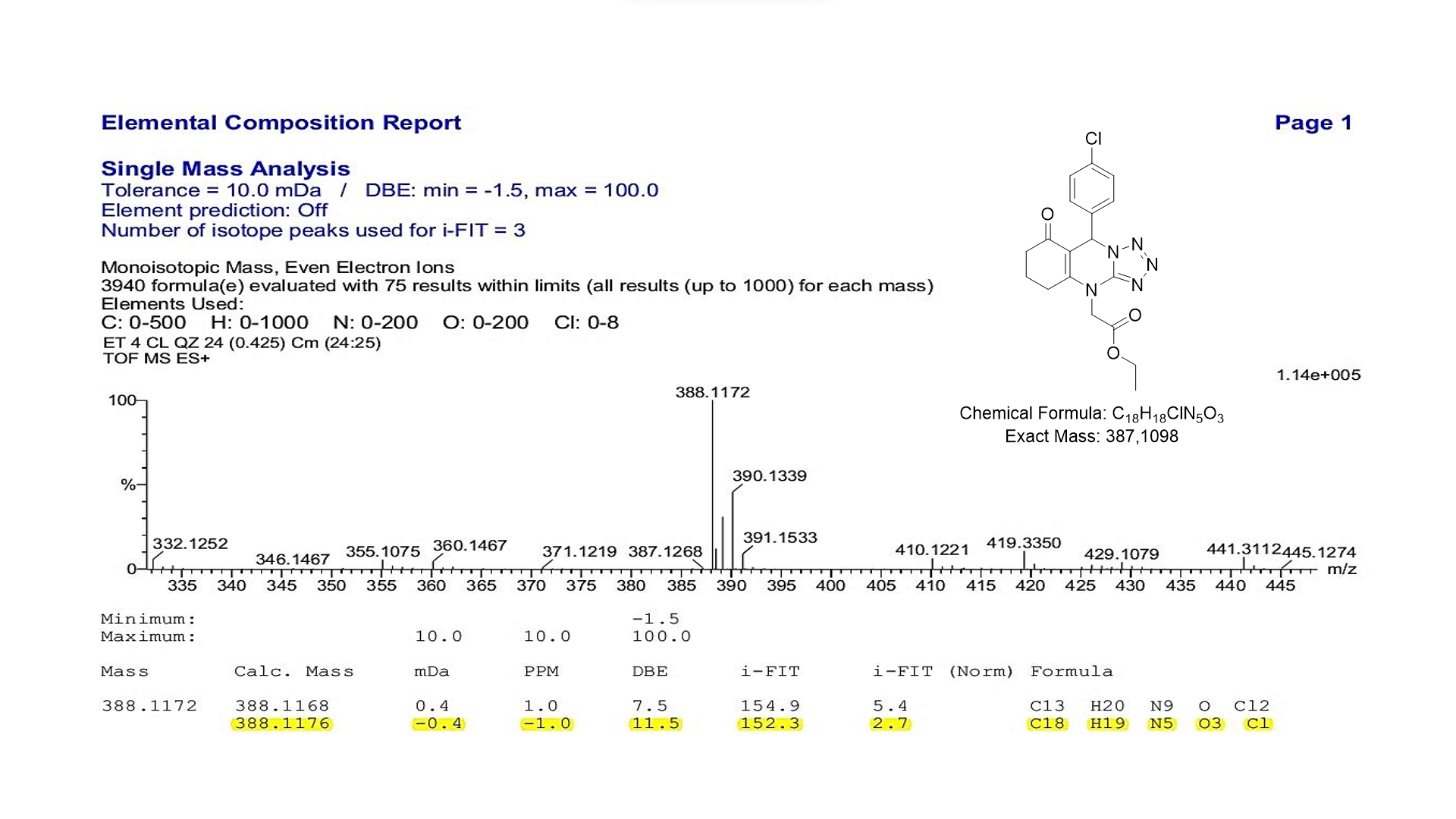


**Figure SM12**. HRMS spectrum of compound **6**

All the spectral analysis was performed in National research and innovation agency (BRIN) in Jakarta, Indonesia.
